# Ectromelia Virus Disease Characterization in the BALB/c Mouse: A Surrogate Model for Assessment of Smallpox Medical Countermeasures

**DOI:** 10.3390/v8070203

**Published:** 2016-07-22

**Authors:** Jennifer Garver, Lauren Weber, Eric M. Vela, Mike Anderson, Richard Warren, Michael Merchlinsky, Christopher Houchens, James V. Rogers

**Affiliations:** 1 Battelle Biomedical Research Center, West Jefferson, OH 43162, USA; weberl@battelle.org (L.W.); andersonms@battelle.org (M.A.); rogersjv@battelle.org (J.V.R.); 2 Vaccine and Gene Therapy Institute, Oregon Health and Science University, Portland, OR 97239, USA; vela@ohsu.edu; 3 Biomedical Advanced Research and Development Authority, US Department of Health and Human Services, Washington, DC 20201, USA; richard.warren@hhs.gov (R.W.); michael.merchlinsky@hhs.gov (M.M.); christopher.houchens@hhs.gov (C.H.)

**Keywords:** ectromelia, BALB/c, smallpox, surrogate model, vaccine, medical countermeasure, antiviral

## Abstract

In 2007, the United States– Food and Drug Administration (FDA) issued guidance concerning animal models for testing the efficacy of medical countermeasures against variola virus (VARV), the etiologic agent for smallpox. Ectromelia virus (ECTV) is naturally-occurring and responsible for severe mortality and morbidity as a result of mousepox disease in the murine model, displaying similarities to variola infection in humans. Due to the increased need of acceptable surrogate animal models for poxvirus disease, we have characterized ECTV infection in the BALB/c mouse. Mice were inoculated intranasally with a high lethal dose (125 PFU) of ECTV, resulting in complete mortality 10 days after infection. Decreases in weight and temperature from baseline were observed eight to nine days following infection. Viral titers via quantitative polymerase chain reaction (qPCR) and plaque assay were first observed in the blood at 4.5 days post-infection and in tissue (spleen and liver) at 3.5 days post-infection. Adverse clinical signs of disease were first observed four and five days post-infection, with severe signs occurring on day 7. Pathological changes consistent with ECTV infection were first observed five days after infection. Examination of data obtained from these parameters suggests the ECTV BALB/c model is suitable for potential use in medical countermeasures (MCMs) development and efficacy testing.

## 1. Introduction

Variola virus (VARV), the causative agent of smallpox, was responsible for over 500 million deaths in the 20th century, with mortality rates for the severe form of smallpox, variola major, greater than 30%. The eradication of smallpox as a human disease was achieved in the 1970s through a concerted vaccination campaign spearheaded by the World Health Organization (WHO) relying on case identification, contact tracing, and focused “ring” vaccination campaigns to stop disease spread [1]. The success of this campaign existed in part because there were no identifiable animal reservoirs of the disease and the highly effective nature of the vaccine, rapidly providing immunity after a single application. However, the evidence that some countries manufactured smallpox as a bioweapon, the possibility that unknown sources of the virus persist [2], and the susceptibility of the United States population to smallpox led the United States Government (USG) to reinvest in the development of smallpox countermeasures, resulting in the approval of a new smallpox vaccine (ACAM2000; Acambis Inc., Cambridge, MA, USA) in 1997 [3]. Although vaccines are extremely effective when administered prior to exposure, the ability of the vaccine to protect against mortality drops rapidly after emergence of the disease and appears to have no ameliorative effect after smallpox symptoms are present. In order to provide treatment options for patients already exposed to smallpox, the USG is investing in the development of small molecule antivirals [4].

In 2007, the Food and Drug Administration (FDA) issued guidance concerning animal models for testing the efficacy of medical countermeasures (MCMs) against VARV, the etiologic agent for smallpox. The FDA directed that antiviral activity should be first evaluated in vitro, prior to in vivo studies. Since clinical trials with smallpox are impossible, the FDA is evaluating the efficacy of countermeasures against smallpox under the FDA Animal Rule (21 CFR 314.600 for drugs and 21 CFR 601.90 for biological products), where animal models are used in lieu of traditional clinical trials to evaluate efficacy. As there are no animal reservoirs for smallpox, there are no susceptible species that can be infected with variola to test the activity of countermeasures. In order to solicit input for identification of models for evaluating smallpox MCMs, the FDA Center for Drug Evaluation and Research (CDER) held an Antiviral Drugs Advisory Committee Meeting to address smallpox concerns in December 2011, resulting in guidance specifying that rabbits challenged with rabbitpox virus (RPXV) and mice challenged with ectromelia virus (ECTV) were acceptable models for product testing. In order to evaluate the relevancy of these models for evaluation of therapeutic efficacy against smallpox, complete characterization of the model to determine the course of disease and specific biomarkers resulting from infection should be identified. Similarly, in human smallpox, a patient develops high fever and characteristic lesional rash after infection, providing clear, unambiguous signs for medical intervention [1]. As studies using rabbit or mouse models had measured the efficacy of a drug or countermeasure when administered based on a delay after exposure to infection, it was not immediately evident if these models would provide symptoms appropriate for countermeasure intervention.

Ectromelia is a member of the genus *Orthopoxvirus,* which includes VARV, vaccinia virus, monkeypox virus (MPXV), and RPXV. ECTV is responsible for mousepox disease and the original Hampstead strain was isolated approximately 85 years ago in a laboratory mouse colony [5]. Since this discovery, various ECTV strains have been isolated from naturally-occurring outbreaks with differences in disease severity. ECTV is infectious at low doses and readily transmitted among wild and laboratory mice populations [6]. The pathogenesis of ECTV infection and the availability of resistant and susceptible mouse strains to ECTV infection has led to many studies investigating the role of protection against infection in the host immune system and the pathogenesis of viral infection [6,7]. Genetic and disease presentation similarities between ECTV and VARV have led to the use of ECTV infection in mice as a laboratory model to test smallpox countermeasures [8,9,10]. As previously reported, the first signs of overt disease, such as lethargy and ruffled fur, normally occur 3–6 days post-challenge in the animals infected with high ECTV titers and 6–8 days post-challenge in animals infected with low ECTV titers [11]. Primary viremia occurs in the mouse after localized replication in regional lymph nodes, resulting in infection of the liver and spleen [12,13]. Mousepox disease severity is largely dependent on both mouse and virus strain, along with the route of infection [7,9].

The ECTV model preferred by the FDA for product testing would resemble smallpox in humans as having high mortality, a low infectious dose, and disease progression through the respiratory system. The ECTV model that best recapitulates these characteristics is the intranasal (IN) challenge model [14] using BALB/c mice at low infectious doses [15]. Therefore, the objective of this study was to provide data supporting use of an IN ECTV challenge in the BALB/c mouse model to test the efficacy of anti-viral MCMs. In this study, a well-characterized stock of ECTV was produced and further characterized in vivo with a natural history of infection following IN challenge, including clinical and pathological response to infection. This model is sufficiently well-characterized to provide data supporting the efficacy of MCMs and dose justification.

## 2. Materials and Methods

### 2.1. Cell Lines

VERO E6 (African green monkey kidney epithelial) cells (NR-596) were previously obtained (BEI Resources, Manassas, VA, USA) and utilized for viral propagation and plaque assay analysis. Cells were grown in Eagle’s Minimum Essential Medium (EMEM; Thermo Fisher Scientific, Pittsburgh, PA, USA) containing 10% fetal bovine serum (FBS; Thermo Fisher Scientific, Gibco, Grand Island, NY, USA) and antibiotics (100 IU/mL penicillin, 100 µg/mL streptomycin, and 0.25 µg/mL amphotericin B; MP Biologicals, Santa Ana, CA, USA). Approximately 3.0 × 10^5^ cells/mL were seeded on 12-well plates (Corning Life Sciences, Corning, NY, USA) and grown to >90% confluence for plaque assay.

### 2.2. Viral Material

Ectromelia virus Moscow strain (ATCC VR1374; American Type Culture Collection, Manassas, VA, USA), designated ECTV-Mos, was propagated in VERO E6 cells for use in this study. Cell lines were determined to be negative for the presence of *Mycoplasma* (Cambrex Corporation, Walkersville, MD, USA) prior to infection. Virus was harvested from the cellular monolayer at a multiplicity of infection (MOI) of 0.01, purified through a sucrose cushion [16], concentrated, tested for sterility, presence of *Mycoplasma* (Cambrex Corporation, Walkersville, MD, USA), endotoxin (Charles River Laboratories, Charleston, SC, USA) and pH levels, and sequenced. The hemagglutinin (HA) gene sequence was 100% identical to the published reference sequence (SeqWright Genomic Services, Houston, TX, USA). ECTV-Mos was diluted in Dulbecco’s phosphate-buffered saline (DPBS) and material was maintained on wet ice following preparation. Confirmation of viral titer post-infection (PI) was determined by plaque assay.

### 2.3. Animals

BALB/c mice (*Mus musculus*, 6–8 weeks of age), free from clinical signs of disease upon arrival as determined by a board-certified veterinarian, were purchased (Charles River Laboratories, Wilmington, MA, USA). Mice were group-housed in disposable, filter-top cages and fed commercial certified rodent chow and water ad libitum. All animal procedures and care were approved by the Institutional Animal Care and Use Committee (Battelle Memorial Institute, Columbus, OH, USA).

### 2.4. Lethal Dose

A dose range study was conducted to determine the lethal dose, 50% (LD_50_) and 90% (LD_90_) of ECTV-Mos in BALB/c mice. Five groups of BALB/c mice, *n* = 16 (8 males/8 females) per group, were inoculated with 2.44, 27.5, 119.5, 292.5, and 700 PFU ECTV-Mos. A probit regression analysis with a base-10 logarithm of dose and mortality as the response was conducted on the survival data.

### 2.5. Natural History of ECTV Infection Following IN Challenge of BALB/c Mice

#### 2.5.1. Study Design

A total of one-hundred eighty BALB/c mice (90 males/90 females) were randomized by weight into 35 groups (Table 1) and inoculated IN at a target dose of 200 PFU (actual dose 125 PFU) ECTV-Mos while under anesthesia. Virus inoculation volume was divided in half and approximately 12.5 µL of virus was introduced per nostril. The uninfected control group received DPBS in the same inoculation volume. Animals in Groups 34 (4 female/4 male) and 35 (4 female/4 male) were designated for mortality assessment at several collection points (Table 1).

#### 2.5.2. Body Weight and Temperature Collection

All animals were weighed prior to infection to determine a baseline. Animals were weighed every other day until succumbing to disease or the end of study on day 14 PI. Body weight was reported as the change from baseline. During the quarantine period, temperature transponder chips (Bio Medic Data Systems, Seaford, DE, USA) were implanted in the rump/hip area on each animal and temperatures were obtained twice a day (AM and PM) from 4 days pre-infection through day 14 PI.

#### 2.5.3. Clinical Observations

Mice were observed three times daily PI for 10 days, followed by twice daily observations until the end of study on day 14. Noted signs of illness included, but were not limited to, mortality, lack of grooming, lethargy, lacrimation, respiratory abnormalities, and lesion progression.

#### 2.5.4. Viremia and Viral Titers

The progression of mousepox disease was followed by serially euthanizing (Table 1, right column) mice and harvesting blood and tissues to determine the viral burden by plaque assay and quantitative polymerase chain reaction (qPCR). Viral load was also measured by obtaining oropharyngeal secretion samples with swabs (Puritan Medical Products, Guilford, ME, USA) [17]. Following collection, individual swabs were placed in 1 mL of 1X phosphate-buffered saline (PBS; GE Healthcare, HyClone Cell Culture, Logan, UT, USA) for viral recovery.

For qPCR analysis, each nucleic acid sample was assayed in duplicate for detection of a portion of the HA gene using a 7900HT RT PCR system (Applied Biosystems, Life Technologies Corp., Carlsbad, CA, USA). Each 25 µL reaction contained 5 μL of sample, TaqMan^®^ Gene Expression Master Mix (Applied Biosystems), sterile nuclease-free water, and a custom gene expression assay (Applied Biosystems) consisting of primers and a 3′-minor groove binding probe specific for a portion of the *HA* (*J7R*) gene [18]. The final primer and probe concentrations were 900 nM and 250 nM, respectively. Thermal cycling conditions were 50 °C for 2 min and 95 °C for 10 min, followed by 45 cycles of 95 °C for 15 s and 60 °C for 1 min. A reference standard consisting of a pre-determined target template concentration was used to construct a standard assay curve. The mean threshold cycle (C_T_) precision between replicate samples was less than 1.0 for sample acceptance. The negative control utilized a set of wells on each plate without the DNA target template and was used to verify amplification. The limit of quantitation (LOQ) is the lowest number of gene copies (gc) in a sample that can be quantitatively determined with suitable precision and accuracy. Prior validation testing determined the lower LOQ value for the assay as 6.85 gc/μL in tissues. To include the isolation process with the PCR results, the following equation was used for the estimated LOQ: 6.85 gc/μL (40 pL/200 μL) × 1000, with 1.37 × 10^3^ gc/mL in blood and assumed 200 μL sample volume eluted as 40 μL of purified total nucleic acid. A similar equation was used for tissue samples, accounting for the weight of the tissue and reported as gc/g of tissue. The reportable value assigned to unknown test samples with a mean concentration of less than the LOQ is referred to as < LOQ.

For plaque assay, serum and processed tissue samples were serially diluted in EMEM containing 1% FBS (GE Healthcare, HyClone Cell Culture, Logan, UT, USA) and 0.1% Gentamycin Sulfate (MP Biologicals, Santa Ana, CA, USA). Viral suspensions of 0.1 mL were inoculated on the cell monolayer in triplicate and adsorbed for 1 h at 37 ± 2 °C, 5% CO_2_ with rocking, followed by an overlay of 1.89% methyl cellulose (Sigma-Aldrich, St. Louis, MO, USA) in 2X EMEM (Quality Biological Inc., Gaithersburg, MD, USA) with 10% FBS, non-essential amino acids (Corning Life Sciences, Corning, NY, USA), and 1% antibiotics. The infected monolayers were incubated for 7 days at 37 ± 2 °C, 5% CO_2_, followed by visualization of plaques stained with a 1.3 g/L crystal violet solution containing formalin. Acceptance of data is established from a previously determined range of 5 to 150 plaques per well. The assay limit of detection (LOD) is less than 10 viral particles per mL.

#### 2.5.5. Hematology and Clinical Chemistry

Whole blood samples were analyzed on a validated ADVIA^®^ 120 analyzer (Siemens Healthcare Diagnostics, Tarrytown, NY, USA) for total white blood cell count (3.20–12.70 × 10^3^/µL), red blood cell (RBC) count (7.00–10.10 × 10^6^/µL), hemoglobin (11.8–14.9 g/dL), hematocrit (36.7%–46.8%), mean corpuscular volume (42.2–59.2 fL) and hemoglobin (13.8–18.4 pg), platelet count (766–1657 × 10^3^/µL), meant platelet volume (5.0–8.0 fL), neutrophil and lymphocyte count with ratio, and monocyte/eosinophil/basophil/differential leukocyte counts.

Serum samples were analyzed on a validated ADVIA^®^ 1200 system (Siemens Healthcare Diagnostics) for blood/urea/nitrogen (BUN), creatinine, BUN/creatinine ratio alanine and aspartate amino transferase, and C-reactive protein (CRP).

#### 2.5.6. Pathology

Complete gross necropsy was performed on mice from Groups 1 through 16, 34, and 35. Spleen and liver were formalin-fixed and processed for hematoxylin-and-eosin (HE) staining using methods described elsewhere [19]. Tissue sections were examined by a board-certified veterinary pathologist. Gross and microscopic diagnoses were entered into the Next Generation PATH/TOX SYSTEM (version 1.7.2.) for data tabulation and analysis.

#### 2.5.7. Statistical Analysis

Survival rates were estimated by Kaplan–Meier curves for each group. Baseline data were used as the endpoint assessment for each parameter. Descriptive statistics were produced for each animal at each sample collection time. Mean changes compared to baseline using analysis of variance (ANOVA) models to evaluate change in status and estimate time to abnormal. Abnormal was defined as the first occurrence of viremia/viral titers, increase in temperature, decrease in body weight, increase/decrease from threshold for hematology and clinical chemistry parameters, detection of a CRP level greater than or equal to the LOD, and the first occurrence of a mild, moderate, or severe clinical observation as determined by pre-defined classification scores in animals following challenge (Table 2). Clopper–Pearson 95% confidence intervals (CIs) were calculated for the proportion of animals defined as abnormal.

## 3. Results

### 3.1. Determination of ECTV-Mos Lethal Dose by Intranasal Challenge

To establish the dose of ECTV stock virus at which 50% or 90% of animals succumbed to disease, five groups of mice (*n* = 16) were challenged intranasally with a target of 5 to 1000 PFU (Table 3).

**Figure 1:**
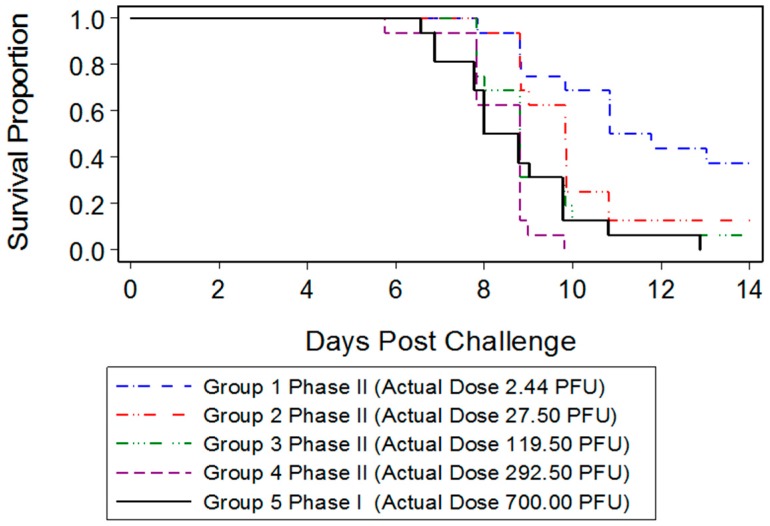
Lethal dose (LD) range survival data for determination of LD_50_ and LD_90_. The mean time-to-death ranged from 8.0 to 10.84 days post-infection (PI) with 700 and 2.44 PFU ECTV-Mos, respectively.

Deaths were first observed six days PI, with all mice in the highest dose group (700 PFU) dying by day 13 following challenge (Figure 1). The LD_50_ was less than 2.44 PFU (95% CI of 0.02, 4.43), assigned as a result of greater than 50% of the mice dying at the lowest challenge dose. The LD_90_ was 32.10 PFU (95% CI of 10.63, 227.47).

### 3.2. Natural History of Infection

#### 3.2.1. Mortality Assessment

**Figure 2:**
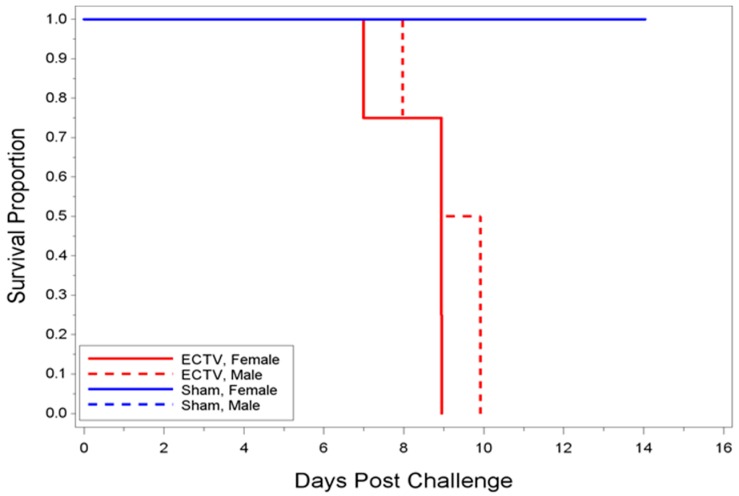
Kaplan–Meier Plot for ECTV-Mos and sham animals. Two groups were assessed for data (*n* = 8 per group). All animals infected with ECTV-Mos succumbed to disease or were euthanized by 10 days PI. All mice inoculated with Dulbecco’s phosphate-buffered saline (DPBS) survived until the end of study.

Kaplan–Meier survival curves were plotted and survival rates summarized by group (Figure 2). All eight ECTV-infected animals in Group 34 succumbed to disease prior to 10 days PI in which the median time-to-death was 8.94 days. No statistically significant difference (*p*-value = 0.3975) of median time-to-death was observed between males (9.43 days) and females (8.94 days). All eight uninfected, sham-inoculated animals survived through the end of the study.

#### 3.2.2. Body Weight

**Figure 3:**
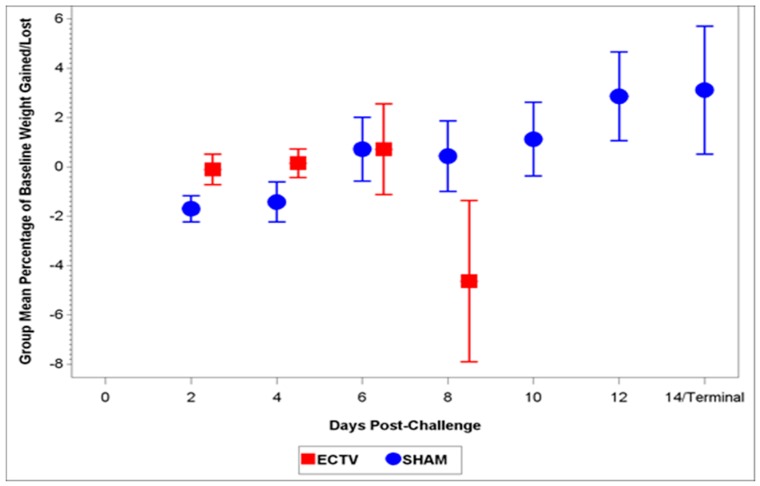
Change from baseline weight in BALB/c mice post-infection. Significant weight loss in mice infected with ECTV-Mos was observed at eight days PI. Sham-inoculated, Group 35 mice lost a statistically significant amount of weight on day 2 and day 4 PI compared to baseline weights; however, these mice gained weight back by day 6.

The body weights from Group 34 (ECTV-Mos) and 35 (sham) were analyzed for statistical comparison as these animals did not undergo serial euthanasia. All except one of the Group 34 mice lost weight prior to death, with significant weight loss occurring at day 8 PI. ECTV-Mos infected mice lost approximately 5% body weight when compared to baseline (Figure 3). Sham-inoculated mice did not experience a significant weight change.

#### 3.2.3. Body Temperature

**Figure 4:**
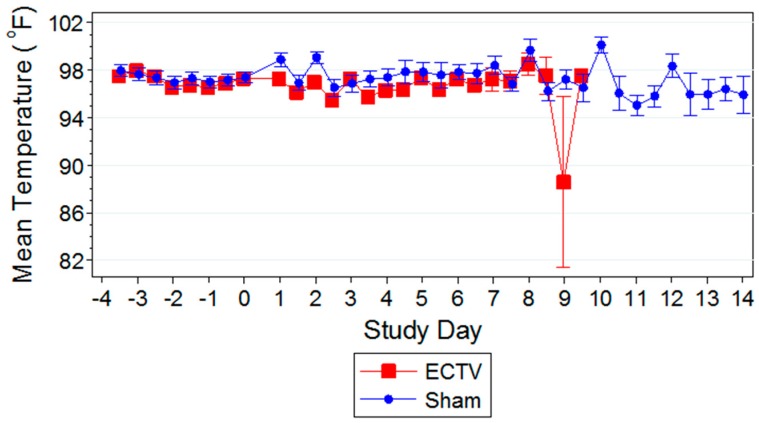
Temperature (°F) with 95% confidence intervals for ectromelia virus (ECTV) and sham mice. Statistically significant changes compared to baseline occurred at random intervals following infection, however were not significant between groups until nine days PI. On study day 9, a dramatic drop in mean temperature was observed in the ECTV-Mos infected group.

Changes in body temperature were observed following ECTV-Mos infection compared to the sham-inoculated mice (Figure 4).

Statistically significant changes occurred in both the ECTV-Mos-infected group and sham-inoculated group when compared to baseline temperatures. Typically, these changes were not remarkable when compared between the two groups. The reduction in mean body temperature of ECTV-Mos-infected mice observed nine days PI was a result of animals exhibiting a reduction in temperature that preceded euthanasia or death.

#### 3.2.4. Clinical Observations

Adverse clinical signs of disease were noted in all ECTV-Mos challenged animals and were significantly different four days PI in comparison to sham controls. Signs of disease consisted of lethargy, ruffled fur, hunched posture, lacrimation, abnormal breathing, labored breathing, ocular abnormalities such as clear discharge from one or both eyes and partial closure of one or both eyes, and lesions observed at the base of the ears and tails. Ruffled fur and lethargy were the most common signs of disease that were first exhibited. In some cases, mild ocular abnormalities (lacrimation) were noted as early as three days PI; however, this was not consistent among all animals. Dyspnea was first observed in ECTV-infected animals seven days following infection. Some animals demonstrated severe general appearance and ocular abnormalities at five and seven days PI, respectively. No adverse signs of disease consistent with ECTV infection were present in the sham control groups.

### 3.3. ECTV Disease Progression

#### 3.3.1. Viral Titers via qPCR

**Figure 5:**
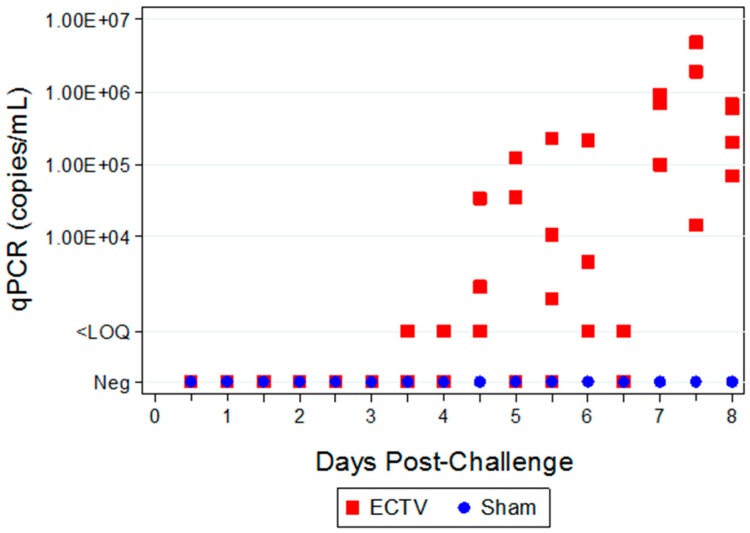
Quantifiable qPCR titers in the blood of ECTV-Mos infected mice were first measured at 4.5 days PI with titers ranging from 2.04 × 10^3^ to 3.39 × 10^4^ gene copies/mL. Viremia was measured in most animals starting at 5.5 days PI and viral genomic material in the blood increased through 7.5 and eight days PI.

Positive detection of ECTV genetic material via qPCR in blood samples first occurred at 84 h PI (Figure 5). However, copies of genetic material were below the LOQ for the assay (< 2.0 copies/reaction). Titers were measured from approximately 10^3^ to 10^6^ gc/mL at 4.5 to eight days PI, respectively (Figure 5).

Approximately 13.5% of the oropharyngeal swabs analyzed for viral genetic material were positive by qPCR analyses, although the number of gene copies in all positive samples were below the LOQ (214 gc/mL). In these cases, viral genomic material in swabs was present between four and eight days PI.

**Figure 6:**
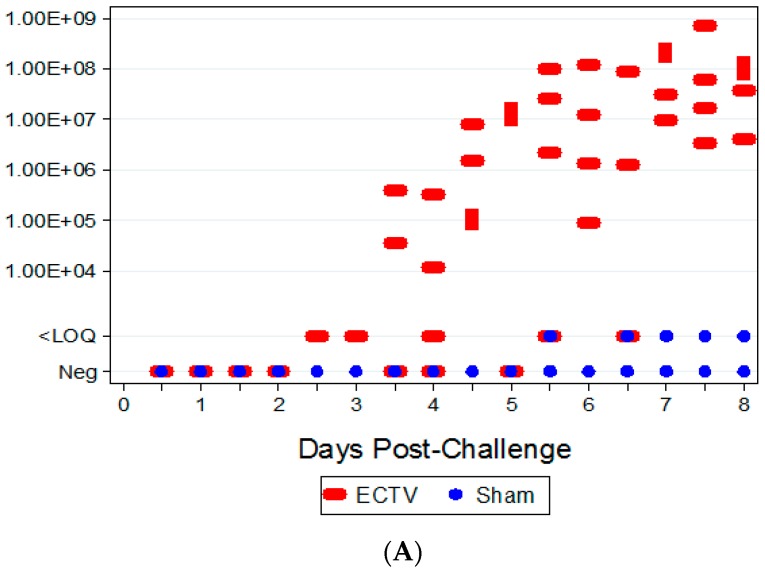

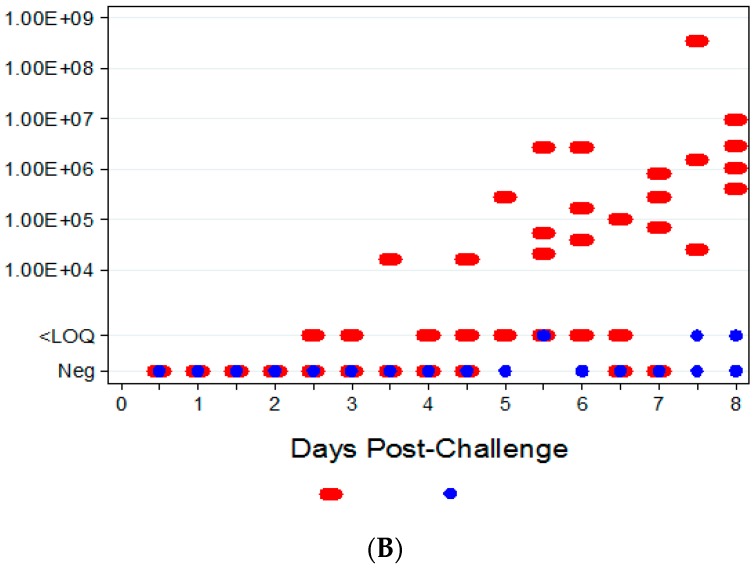
Animal qPCR titers in tissue. (**A**) qPCR titers in the spleen following ECTV-Mos infection. Quantifiable viral titers were first measured at 3.5 days and increased as time progressed, with the highest titers present at seven to eight days following challenge; (**B**) qPCR titers in the liver following ECTV-Mos infection. Viral titers were first measured at 3.5 days PI and slightly increased over time, with the highest titers observed between 7.5 and 8 days following challenge.

The presence of the virus was observed in the spleen by 2.5 days PI via qPCR; however, the number of gene copies detected from these samples were below the assay LOQ. Quantifiable viral titers were first measured in spleen samples from ECTV-Mos-infected mice at 3.5 days PI (Figure 6A). As a whole, viral titers in the spleen increased in the animals that were serially-euthanized after 4.5 days PI. Similar results were observed in the liver (Figure 6B); however, titers in the liver were lower when compared to the titers in the spleen. ECTV was not detectable by qPCR in the blood, oropharyngeal swabs, and harvested spleen and liver tissues from sham-inoculated mice.

#### 3.3.2. Viral Titers via Plaque Assay

**Figure 7:**
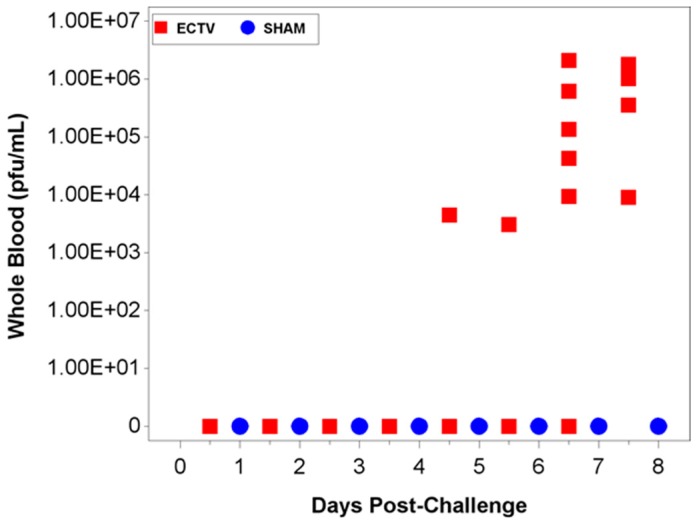
Viremia as measured by plaque assay. The first positive viremia titers via plaque assay occurred at 4.5 days following ECTV-Mos infection. Titers varied from 10^3^ (4.5 days PI) to 10^6^ PFU/mL (7.5 days PI).

All blood samples taken from animals following ECTV-Mos infection were positive for viremia via plaque assay (Figure 7). When evaluating blood samples obtained at each scheduled collection, approximately 25% of the samples were positive for viremia via plaque assay analysis. However, not every sample was assayed (as a result of low collection blood volumes).

**Figure 8:**
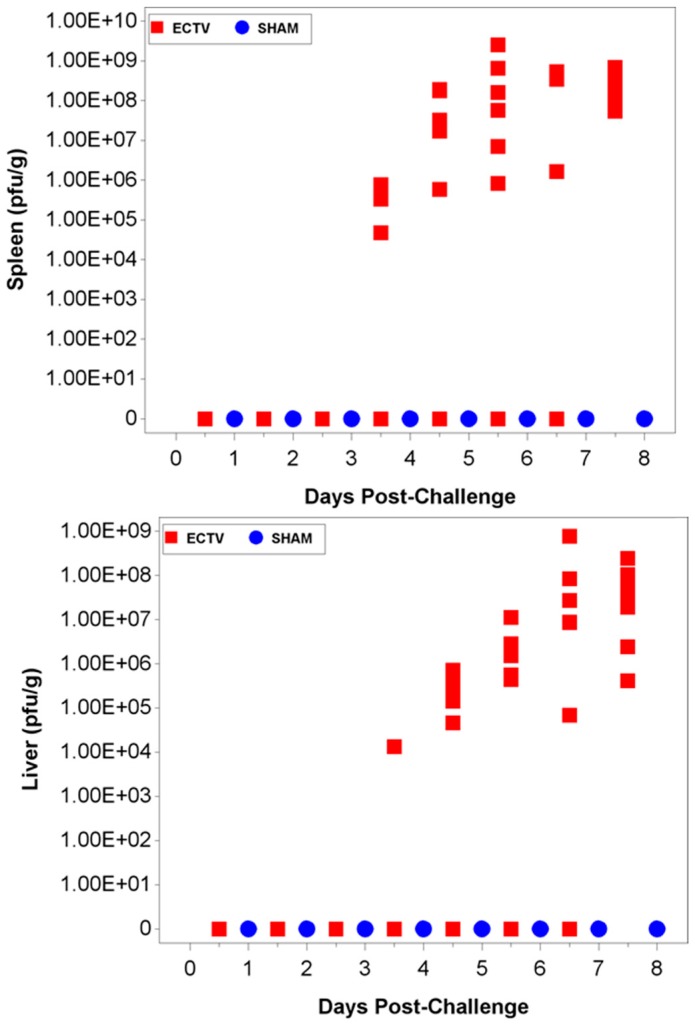
Viral load in tissues as measured by the plaque assay. (**A**) the first positive viral titer displayed in the spleen was observed at 3.5 days PI. Titers ranged from 10^4^ to 10^9^ PFU/g up to 7.5 days PI; (**B**) viral titers in the liver were first observed at 3.5 days PI. Titers ranged from 10^4^ to 10^9^ PFU/g up to 7.5 days PI. Approximately 76% of liver samples collected at 3.5 days PI or later were positive for virus via the plaque assay.

When evaluating spleen samples at 3.5 days PI, approximately 82% of the spleen samples were positive for viral titers via plaque assay analysis (Figure 8A). Overall, viral load in the spleen was higher during later collection points with respect to challenge. Similarly, the first positive liver sample via plaque assay also occurred 3.5 days PI in the same animal that was positive at the same qPCR collection time (Figure 6B and Figure 8B).

#### 3.3.3. Hematology and Clinical Chemistry

**Figure 9:**
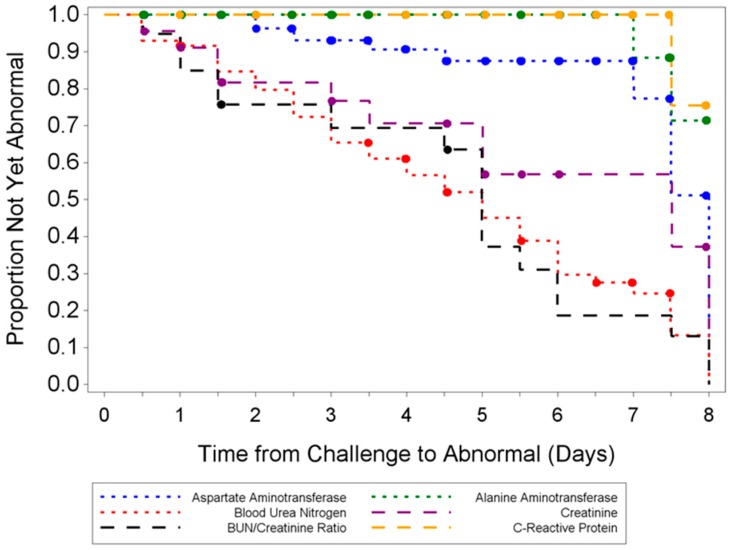
Kaplan–Meier curve showing abnormal clinical chemistry parameters for ECTV-infected animals. Blood/urea/nitrogen (BUN) and BUN/creatinine ratio levels yielded abnormal results in the majority of evaluated animals (50% or greater), where levels were abnormally high (normal BUN levels, 17.0–60.0 mg/dL). A proportion of the animals demonstrated abnormal AST (15%) and ALT (3%) levels, while 43.5% of animals demonstrated abnormal creatinine levels.

Several measured hematology parameters yielded abnormal results in >50% of animals challenged with ECTV-Mos. Parameters with abnormal results included RBC count, lymphocyte count, monocyte count, basophil count, and large unstained cell count. Although these parameters were abnormal in >50% of sampled animals, there were no distinguishable trends. For clinical chemistry, data demonstrate notable levels of liver and kidney abnormalities in some of the animals infected with ECTV-Mos (Figure 9).

#### 3.3.4. Pathology

Gross observations from animals exposed to ECTV-Mos included pocks on the tail, discoloration of the spleen, and discoloration of the liver. Gross findings in the spleen and liver corresponded microscopically to hemorrhage and necrosis, respectively, both of which are considered consistent with the effects of ECTV infection. Lesions typical of ECTV infection included lymphoid necrosis in the white pulp of the spleen that varied from multifocal at the earlier time points, to diffuse throughout the white pulp at later time points. Aggregates of fibrin and variable numbers of inflammatory cells (neutrophils and macrophages) were admixed with necrotic lymphocyte cell debris. Necrosis extended into the red pulp in the most severely affected cases, often leaving only hemorrhages with scattered aggregates of necrotic cell debris and fibrin. In the liver, early lesions consisted of rare, scattered foci of hepatocellular necrosis, usually affecting five to eight contiguous hepatocytes. Cytoplasmic inclusions typical of such an infection were consistently present in adjacent non-necrotic hepatocytes. At approximately six days following infection, the foci of necrosis became more widespread, eventually affecting up to 50% of the examined liver section. There were no findings of gross or microscopic observations consistent with ECTV-Mos infection in the sham control groups.

## 4. Discussion

The regulatory evaluation of MCMs against smallpox requires the use of animal models challenged with the cognate poxvirus to serve as surrogates for natural VARV infection in humans. The countermeasure intended for therapeutic intervention requires an animal model with unambiguous and reliable symptoms of the disease to serve as triggers for intervention. Although scientific literature contains many publications demonstrating the ability of drugs and antibodies to block poxvirus infections, studies are typically described in which drug intervention is based on a time delay after exposure. These models of post-exposure prophylaxis reflect a scenario where a drug is given after a putative exposure of virus, prior to disease symptoms.

Prior to the WHO Advisory Committee Meeting on VARV in 2012, the only animal model used in the therapeutic evaluation of smallpox countermeasures was the MPXV intravenous challenge of cynomologus macaques [20]. Although intervention in numerous MPXV studies was based on time delay, investigators were consistently able to document that all animals exhibited symptoms of disease (temperature elevation, viremia) prior to drug intervention [21]. Since regulatory evaluation of smallpox countermeasures would require demonstration of effectiveness in multiple animal models, two animal models proposed by the FDA for development of a therapeutic model for countermeasure evaluation include the New Zealand White rabbit infected with RPXV and the BALB/c mouse infected with ECTV.

Inconsistencies with mouse strain variation, titer determination, and inoculation route in previous studies have led to difficulties in developing an effective treatment model [22]. In this study, we describe an investigation into the ability of the IN BALB/c mouse challenge model to serve as an animal model to evaluate the therapeutic efficacy of smallpox countermeasures. Data were collected and biomarkers evaluated for suitability of use for clinical intervention to test the therapeutic efficacy of MCMs for human smallpox disease. The characterization of ECTV infection was investigated in this study through delineation of viral potency, lethal infective dose, mortality, alterations in temperature and weight, viral burden, pathological changes, and clinical observations. There were no unambiguous, reproducible biomarkers associated with infection until late in disease progression in which the earliest and most reliable indicator of ECTV infection was the accumulation of replicating virus in the liver and spleen, consistent with earlier observations in ECTV-infected mice [8]. The concentration of viruses in the liver and spleen may indicate tissue necrosis as the primary causes of death, differing from humans infected with smallpox as hepatic and splenic necrosis are not considered primary causes of mortality [8,9].

The ECTV murine model mimics many features similar to that of human smallpox, including acute systemic disease caused by a low viral infectious dose, lack of pulmonary involvement early in the disease progression, viremia, and fever [6]. A consistent, high mortality rate in BALB/c mice challenged IN with a high dose (10^3^ PFU) of ECTV allows for a disease progression that begins 3–4 days PI and results in complete mortality within 10 days after challenge [23]. In the present study, we were interested in developing a model more reflective of the moderate or low dose exposure needed for natural infection, while retaining high mortality in order to measure the statistical significance of MCM intervention. Mice infected with 700 PFU ECTV-Mos succumbed to disease as early as six days after challenge, with all mice dead by 13 days PI. The mean time-to-death was inversely proportional to the dose as mice infected with 700 PFU (eight days) died sooner than mice infected with a lower PFU (2.44 PFU, 10.84 days). A statistically significant difference in the median time-to-death between males and females following infection could not be determined, indicating the sex of the mouse did not appear to influence mortality.

Classic symptoms routinely observed after high dose challenge in mouse models were also observed. Significant weight loss, commonly noted in high dose challenge models in animals infected with ECTV-Mos, was only initially observed eight days following challenge. Statistically significant body temperature changes were not observed until nine days post-challenge. The appearance of weight loss and temperature changes very late in the disease course suggests these biomarkers may not be useful “trigger-to-treat” parameters used for drug intervention in therapeutic efficacy studies. Historically, temperature change has not been a reliable biomarker as an early indication of ECTV disease infection [17].

Clinical signs of disease including lethargy, ruffled fur, hunched posture, abnormal breathing, labored breathing, lacrimation, ocular abnormalities, and lesions were monitored to determine if an unambiguous indication for disease intervention could be presented. The mild clinical signs, such as lethargy and ruffled fur, were not good indications of disease as they were observed, albeit rarely, in sham infected animals, implying that the identification of these symptoms was not unambiguous for infected animals. The most common lesions appeared on the tail of mice in a small percentage of animals. However, group-housed mice could provoke tail biting, creating difficulty in discerning a pox lesion from a lesion caused by biting. Therefore, it is inconclusive as to whether all lesions observed in this study were directly related to ECTV infection or inflammation. Several mice did exhibit ear lesions that appeared at the base of the ears; however, these lesions were sporadic and relatively uncommon. As fur was not removed from mice, it was difficult to evaluate whether animals exhibited lesions or an ECTV-induced rash on the skin. Thus, utilization of rash or lesions as a potential trigger-to-treat in future mouse studies may be difficult without removal of hair on the BALB/c model. Some hairless mouse strains, such as the SKH1 strain, are sensitive to ECTV when the virus is administered via the IN route [9]. Mouse models utilizing lesion presentation as a treatment trigger may not be advantageous since the presentation of lesions occurs late in the disease process, which, in turn, does not allow for a proper therapeutic window to observe product efficacy. The difference in clinical signs for moderate or severe symptoms between the infected and sham infected animals was significant by day 7 post exposure, just prior to mortality, demonstrating that clinical signs could be used to identify infected animals and potentially serve as triggers for intervention. Since observations consistent with mousepox disease were only apparent very late after infection, using clinical signs to inform drug intervention suggests that the time of intervention in the mouse model will be very late in disease course and not analogous to time of intervention and therapeutic window for smallpox disease in humans.

Two attempts were made to measure disseminated virus after infection. Blood was drawn at specified times and viral copy number (using PCR) and infectious virus (using plaque assay) were measured. In addition, as a non-invasive method that could be adaptable for real-time and multiple observations, oropharyngeal swabs from the oral cavity were collected and tested for viral copy number by qPCR. However, the viral copy numbers were below the assay LOQ in all swab samples from infected animals, with only a small percentage of animals having detectable viral genome copy numbers. Therefore, oropharyngeal swabs are not a reliable biomarker for countermeasure intervention. For blood samples, viral genomic material and infectious virus were observed at quantifiable levels in most, but not all infected animals starting at 4.5 days PI and the values increased with disease progression.

The ability to detect genomic copy numbers and infectious virus in spleen and liver tissue was achieved through qPCR and plaque assay. Quantifiable viral genomic material measured by qPCR and viral titers by plaque assay were first measured in the spleen and liver at 3.5 days PI, increasing in both tissues as the disease progressed. Overall, the level of viral genomic material in the liver was slightly less than observed in the spleen; however, all infected animals had measureable levels of genomic copies. Not surprisingly, the plaque assay for detecting infectious virus was less sensitive than qPCR as fewer ECTV-Mos infected animals exhibited detectable viral titers by plaque assay. In contrast to the ability to detect copy numbers or infectious virus in the blood, which was not universal for infected animals until at least seven days post exposure, splenic tissue samples did reproducibly and unambiguously identify infected animals by 5 to 5.5 days post exposure. Consideration must be given to this method for confirmation of viral detection, as sacrificing the animal to determine the level of infection is an evident drawback of tissue collection.

Hematological and clinical chemistry parameters were measured and screened for statistically significant differences compared to sham-infected animals and baseline. Several parameters demonstrated abnormal results in a majority of infected animals; however, no distinguishable trend for a reliable and unambiguous biomarker was observed. Pathological findings from tissues of challenged animals exhibited microscopic evidence of virus in the organs examined. Gross observations included pocks on the tail and discoloration of the spleen and liver. Tissue lesions were first noted at five days PI. Following ECTV-Mos infection, the time at which animals succumbed to disease correlated with the onset of the most severe microscopic findings.

The aim of these studies was to characterize the ECTV intranasal challenge model in BALB/c mice as a potential animal model for therapeutic evaluation of smallpox countermeasures. The ECTV model resembles human smallpox in that a low respiratory challenge dose produces high mortality. We intended to identify biomarkers to define the appropriate time for product intervention in order to measure therapeutic efficacy. The onset of clinical signs confirming ECTV infection are apparent relatively late in the course of the disease and do not provide a therapeutic window suitable for product evaluation. The easily measured and non-invasive analyses of weight loss, temperature rise, and genomic copies from oropharyngeal swabs are not reliable and unambiguous until just prior to death. The biomarker with the earliest indication of ECTV infection is genomic copy number as measured by qPCR. These copy numbers are detectable as early as at day 2.5 (organs) and 3.5 (blood). The percentage of infected animals with high copy numbers increases until all animals are positive by days 6 (spleen) or 7 (blood).

In human smallpox there is an extended incubation period (7–17 days) followed by prodromal fever and eruption of lesions, with patient outcome decided approximately two weeks after prodrome [21]. In the mouse, the analogous disease caused by ECTV is compressed with disease progression and outcome completed in about one and a half weeks [11]. The extended time from exposure to outcome in humans suggests that a biomarker earlier than one or two days prior to death in the mouse model would be a better analogue to the timing of intervention in a smallpox case. The medical intervention in smallpox would ideally occur near the midpoint of the disease course, as soon as lesions are detected, or after an outbreak is confirmed, and with the onset of fever in exposed patients. The biomarker in the mouse model that appears to correspond to this timeline is the PCR detection of viral genomes in blood and organs. However, the use of these biomarkers as triggers for intervention in a blinded study may require ingenuity in protocol design by culling a portion of each group as sentinel animals for disease detection or, as in the case of MPXV experiments, using a time-based intervention with confirmation of infection post-hoc.

The ECTV mouse model has afforded a powerful tool to study poxvirus-host interactions since there are genetic mouse strains that are sensitive and others that are resistant to infection. The cell-mediated arm of the immune response appears to be critical in determining the sensitivity of the mouse to ECTV infection [7,12,24,25]. The ability of the mouse to generate an early and robust cell-mediated immune response to clear the initial infection is the primary factor in surviving ECTV infection in resistant mice. The cell-mediated response limits the virus to the regional lymph nodes, prevents extensive liver and spleen damage, and prevents systemic spread. However, in sensitive strains of mice, such as BALB/c, the lack of an effective cell-mediated response leads to extensive replication in the liver and spleen and, just prior to death, systemic spread [11]. The data from our studies are consistent with these observations. We detected early viral replication in the liver and spleen as well as the upper respiratory tract, while dyspnea and ocular discharges were noted late in infection.

The use of this BALB/c ECTV challenge model provides another method for the evaluation of smallpox countermeasures. The low cost and small size of mice allow for conducting large, statistically powered studies. In addition, use of a BSL-2 virus allows for greater accessibility compared to the rigid select agent practices necessary when working with BSL-3 agents, such as MPXV. Thus, ECTV infection in the BALB/c mouse provides a potential model for assessment of therapeutics and development of effective medical countermeasures of disease.

## Figures and Tables

**Table 1:** Study design of ectromelia virus Moscow strain (ECTV-Mos) characterization in the BALB/c mouse model

Group	Inoculation	No. of Animals (M/F)	Oropharyngeal Secretions Collection Times - Swabs (Hours PI)	qPCR/Plaque Assay Collection Times (Hours)	Hematology/Clinical Chemistry Collection Times (Hours PI)
1	ECTV	4/4	12, 24	12, 24	NA
2	SHAM	2/2			
3	ECTV	4/4	36, 48	36, 48	
4	SHAM	2/2			
5	ECTV	4/4	60, 72	60, 72	
6	SHAM	2/2			
7	ECTV	4/4	84, 96	84, 96	
8	SHAM	2/2			
9	ECTV	4/4	108, 120	108, 120	
10	SHAM	2/2			
11	ECTV	4/4	132, 144	132, 144	
12	SHAM	2/2			
13	ECTV	4/4	156, 168	156, 168	
14	SHAM	2/2			
15	ECTV	4/4	180, 192	180, 192	
16	SHAM	2/2			
17	ECTV	2/2	NA	NA	0
18	ECTV	2/2			12
19	ECTV	2/2			24
20	ECTV	2/2			36
21	ECTV	2/2			48
22	ECTV	2/2			60
23	ECTV	2/2			72
24	ECTV	2/2			84
25	ECTV	2/2			96
26	ECTV	2/2			108
27	ECTV	2/2			120
28	ECTV	2/2			132
29	ECTV	2/2			144
30	ECTV	2/2			156
31	ECTV	2/2			168
32	ECTV	2/2			180
33	ECTV	2/2			192
34	ECTV	4/4			NA
35	SHAM	4/4			

M/F: male/female; qPCR: quantitative polymerase chain reaction; NA: not applicable; ECTV: ectromelia virus; SHAM: sham control; PI: post infection.

**Table 2:** Clinical conditions and definitions.

Severity of Condition	Clinical Condition/Definition
	**General Appearance**
Mild (1)	1 of 3 of the following signs: Lethargy, Hunched Posture, Ruffled Fur
Moderate (2)	2 of 3 of the following signs: Lethargy, Hunched Posture, Ruffled Fur
Severe (3)	3 of 3 of the following signs: Lethargy, Hunched Posture, Ruffled Fur or Lesions present
	**Dehydration**
Mild (1)	Slight decrease in skin turgor, skin will not tent
Moderate (2)	Moderate decrease in skin turgor; skin will tent and slowly (several seconds) return to normal
Severe (3)	Skin visibly wrinkled, dry; greatly decreased skin turgor, skin will tent
	**Dyspnea**
Mild (1)	Undefined
Moderate (2)	Demonstrates respiratory abnormalities
Severe (3)	Demonstrates labored breathing
	**Ocular Abnormalities**
Mild (1)	Clear discharge from one or both eyes
Moderate (2)	Clear discharge from one or both eye; partial closure of one eye
Severe (3)	Clear discharge from one or both eyes; and/or partial closure of both eyes

**Table 3:** Mortality comparison by target dose.

Group	Target Dose (PFU)	Back Titer (PFU)	Number of Animals Succumbed/Total Number of Animals	Mortality Proportion (Binomial Exact 95% Confidence Interval)
1	5	2.44	10/16	0.63 (0.35, 0.85)
2	50	27.50	14/16	0.88 (0.62, 0.98)
3	200	119.50	15/16	0.94 (0.70, 1.00)
4	500	292.50	16/16	1.00 (0.79, 1.00)
5	1000	700	16/16	1.00 (0.79, 1.00)
